# Imprinted Genes: Genomic Conservation, Transcriptomic Dynamics and Phenomic Significance in Health and Diseases

**DOI:** 10.7150/ijbs.83712

**Published:** 2023-06-12

**Authors:** Shane A. Carrion, Jennifer J. Michal, Zhihua Jiang

**Affiliations:** Department of Animal Sciences and Center for Reproductive Biology, Washington State University, Pullman WA 99164-7620, USA.

**Keywords:** Parent of Origin, Comparative Genomics, Gene Variation, Disease Classification

## Abstract

Since its discovery in 1991, genomic imprinting has been the subject of numerous studies into its mechanisms of establishment and regulation, evolution and function, and presence in multiple genomes. Disturbance of imprinting has been implicated in a range of diseases, ranging from debilitating syndromes to cancers to fetal deficiencies. Despite this, studies done on the prevalence and relevance of imprinting on genes have been limited in scope, tissue types available, and focus, by both availability and resources. This has left a gap in comparative studies. To address this, we assembled a collection of imprinted genes available in current literature covering five species. Here we sought to identify trends and motifs in the imprinted gene set (IGS) in three distinct arenas: evolutionary conservation, across-tissue expression, and health phenomics. Overall, we found that imprinted genes displayed less conservation and higher proportions of non-coding RNA while maintaining synteny. Maternally expressed genes (MEGs) and paternally expressed genes (PEGs) occupied distinct roles in tissue expression and biological pathway use, while imprinted genes collectively showed a broader tissue range, notable preference for tissue specific expression and limited gene pathways than comparable sex differentiation genes. Both human and murine imprinted genes showed the same clear phenotypic trends, that were distinct from those displayed by sex differentiation genes which were less involved in mental and nervous system disease. While both sets had representation across the genome, the IGS showed clearer clustering as expected, with PEGs significantly more represented than MEGs.

## Introduction

The field of genomic imprinting has seen rapid advancement since the discovery of the first imprinted gene in 1991, but it was several breakthrough murine experiments in the preceding years that made it possible. Prior observations had found that mammalian, specifically mouse, parthenotes failed to develop to term. At that time there were several theories to explain the phenomenon, the most prominent being abnormal cytoplasm and homozygous lethality. These questions prompted two experiments in 1984, performed by Davor Solter and James McGrath in Philadelphia and Azim Surani, Sheila Barton and Michael Norris in Cambridge 1,2. Both utilized pronuclear transplantation, generating gynogenetic/androgenetic or only gynogenetic embryos respectively, followed by transferring the resulting embryo to a surrogate to carry to term. In each experiment, embryos with two female or two male chromosomes failed to develop post-implantation, with gynogenetic embryos displaying developed core lineages but stunted extraembryonic lineages, and the androgenetic embryos developing normal extraembryonic lineages but deficient core lineages. This revealed two key facts, that cytoplasmic aberrations and homozygotic lethality were insufficient to explain the observed phenomenon, and that despite being *genetically* identical the maternal chromosome and paternal chromosome were clearly not *functionally* identical. The realization that one chromosome from each parent was required for normal development, despite no genetic justification, was one of the primary drivers that ultimately led to the discovery of genomic imprinting.

In 1985, Cattanach and Kirk 3 noted that while both parental chromosomes were required for full embryogenesis, disomy in most chromosomes was demonstrably not lethal, indicative of specific chromosome regions being associated with imprinting effects. Using both reverse and Robertsonian translocations, they were able to identify regions of mouse chromosomes 2 and 11 where uniparental disomy (male and female) resulted in distinct phenotypes, affecting long term viability of the embryo, maternal behavior, and growth. This showed that imprinting effects were clustered and expressed in relatively narrow bands, an important distinction that hinted at both their evolution and method of regulation. The synteny of these bands with human chromosomes would become more and more relevant as similar human conditions were discovered. Finally, in 1991 Barlow et al., utilizing previous efforts to identify the *TME* locus, used systematic deletions and expression analysis to identify *IGF2R* as the first imprinted gene, a maternally expressed and thus paternally silenced gene 4. That same year, DeChiara et al. and Bartolomei et al. were able to show that *IGF2* (paternally expressed), and *H19* (maternally expressed and the first imprinted non-coding RNA (ncRNA)), were also imprinted genes 5,6.

This altered gene expression, a form of parent-of-origin effect not determined by the genetic code, was termed genomic imprinting. Since then, scientists have uncovered a dearth of information about its function and the mechanisms that establish and control it. In eukaryotic species, organisms receive half of their genetic material, or chromosomes, from each parent across all somatic cells. This results in gene expression from both alleles. However, imprinting silences the expression of either the maternally or paternally inherited allele utilizing epigenetic mechanisms, resulting in only one actively transcribed allele and making those genes effectively haploid 7-11. This phenomenon has also proven to be tissue and stage dependent, meaning that a gene could be imprinted in liver tissue (such as *ALDH1L1*) during early stages, but biallelically expressed in later stages and in all other tissues. Even within a single gene, individual transcripts can vary in imprinting status, such as occurs in the gene *GNAS* 12,13.

The functional haploidy of these genes abandons the traditional protections of biallelic expression, the redundancy of two active alleles. In non-imprinted genes, genetic mutations that result in the reduced function or non-function of one allele can be compensated for by the remaining allele, at reduced or sometimes equivalent expression levels. When a mutation occurs in the active allele of an imprinted gene, no such protection exists, leading to a range of diseases and disorders. Several theories have been proposed to explain the evolution of genomic imprinting, the most prominent being the kinship, sexual antagonism, and maternal-offspring coadaptation theories. Though experimental evidence suggests imprinting only occurs in 1-2% of genes, these genes have been increasingly linked to developmental abnormalities, cancers, and mental health concerns 7,10,14. Imprinted genes such as *DLK1* and *NNAT* are associated with gestational complications like preeclampsia and intrauterine growth restriction (IUGR), which can cost 3 times more than a normal birth in pre- to postnatal care 15-19. Births facilitated by assisted reproductive technologies (ART) have also been linked to increased incidence of imprinting disorders, in particular Beckwith-Wiedemann syndrome 20,21.

Since the discovery of imprinted genes more than 30 years ago, significant strides have been made in understanding basic mechanisms, developmental timelines, and categorization of imprinted genes, though a slew of unanswered questions remain. Initial estimates placed the number of imprinted genes in the low hundreds per relevant species, and that estimate has remained fairly stable over the last couple of decades with a few notable exceptions. In 2005, analysis of the FANTOM2 data set provided approximately 2000 candidates for imprinted genes and, as recently as 2010, another group used RNA-seq data in a genome wide analysis that identified 1300 loci with parental expression bias 22-24. While additional studies were done to explain the discrepancy presented by these outlier estimates, they highlight one of the difficulties that continues to affect studies even now, identification and verification of imprinted genes 25. Even between the major imprinting databases, discrepancies exist in which genes are catalogued as well as the imprinting status, literature support, and species represented. Here we assembled a collection of 581 imprinted genes including experimentally validated, predicted and provisional candidates from three primary sources, geneimprint.com 26, igc.otago.ac.nz 27, and a paper by Tucci et al., 2019 28 covering human, bovine, murine, ovine, and porcine genes (Supplemental Table 1). Each gene was noted for species represented (where a human ortholog existed), imprinting status per species and its imprinting status per source. Where there was no human ortholog, the gene was listed with its species-specific gene name and nomenclature. With this collection we review evolutionary features (conservation of orthology, synteny, and biotype), expression patterns (abundance, peak tissues, and cross-tissue trends), and health phenomics (disease, disorders, and defects in human and model organisms). In order to clearly identify defining characteristics, we also reviewed 110 genes (Supplemental Table 2) involved in sex differentiation (so called the sex differentiation gene set (SDGS) 29. Data utilized was primarily taken from NCBI annotations.

## Detecting Imprinted Genes, Past and Future

Papers published as recently as the last decade still continue to note differing numbers of imprinted genes, almost exclusively in the human and murine genomes where the vast majority of imprinting studies have been concentrated due to ease of use and disease similarity between the two species 30,31. This highlights the difficulty of first identifying, then verifying imprinted gene status, which is complicated by the fact that imprinting can be spatially and temporally specific, with very low gene expression in some cases. Fortunately, the methods of capturing and analyzing imprinted gene data have advanced significantly, particularly in the last 5-10 years.

Early study of imprinted genes relied heavily on SNP (single nucleotide polymorphism) arrays and microarrays combined with chromosomal rearrangement techniques, but were limited by cost, availability, inability to detect de novo sequences, and false discovery rate due to alternative transcripts, downstream off-targets and cross-hybridization 32-34. Another time-tested method is locating methylated cytosines through bisulfite sequencing (including whole genome bisulfite sequencing, WGBS) to identify CpG islands and the differentially methylated regions (DMRs) which regulate clusters of imprinted genes. Though WGBS can be expensive and requires a well-documented genome, bisulfite sequencing (particularly new methods such reduced representation bisulfite sequencing) is still used today in combination with other techniques, particularly to validate findings 32,35.

RNA-seq was the next major advancement in sequencing and has been used extensively for whole transcriptome studies, in part because the ease of library creation made it an abundantly available source of data. Study of allele specific expression has been the standard for parsing RNA-seq data in imprinting research and many pipelines have been developed that can be used for this purpose, such as whole transcriptome RNA-seq (WT RNA-seq). While this technique resulted in the discovery of many new imprinted genes, limitations such as a relative lack of complexity in their libraries, maternal contamination in placental tissues, difficulty detecting low levels of expression, and SNPs near junctions/non-polymorphic/in repetitive regions including copy number variants have made it challenging to detect new imprinted genes 25,36,37.

Recent advancements, such as the development of single cell RNA-seq (scRNA-seq), and long read sequencing methods like those provided by PacBio and Nanopore (which allows for detection of allele methylation) have already increased our understanding of imprinted genes, allowing for reads that can overlap multiple relevant SNPs, increasing the accuracy of haplotypes and improving detection of low expression genes 38-40). scRNA-seq also helps to address the problem of epigenetic mosaicism, where individual cells may differ in their imprinted gene expression patterns from mono- to bi-allelic, as well as the spatial and temporal challenges mentioned previously 41.

## Evolutionary Genomics of Imprinted Genes

### Orthologous Dynamics

**Figure 1:**
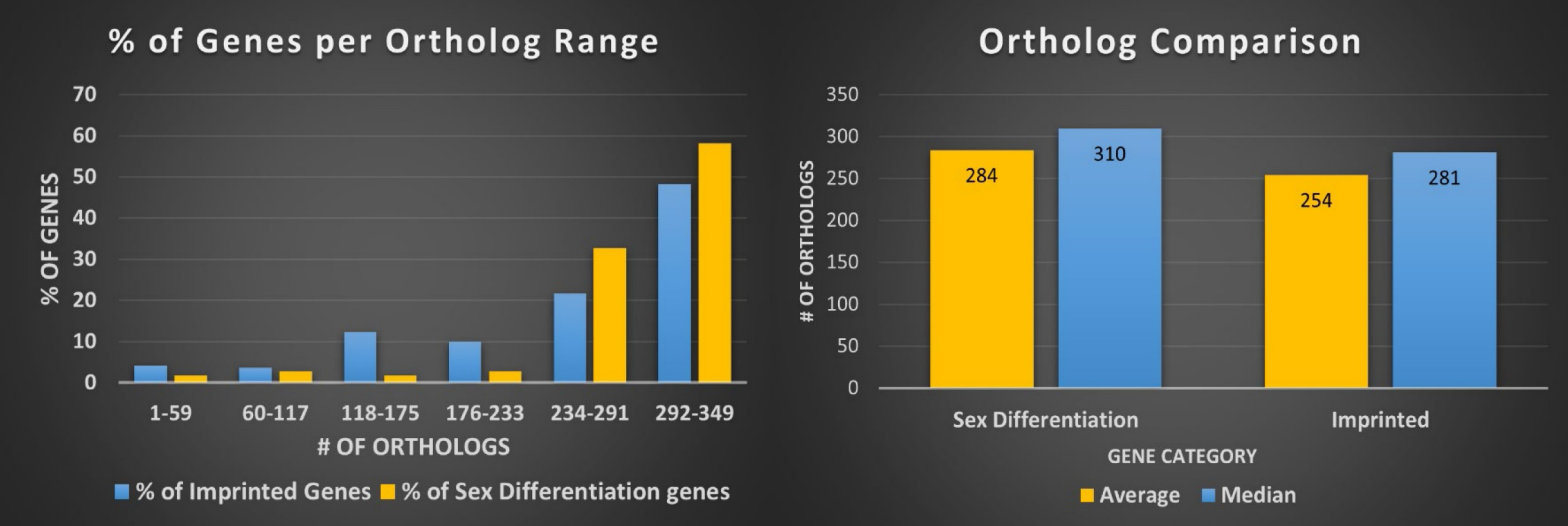
** Imprinted and sex differentiation gene orthologs. (A)** Percentage of orthologs by number of orthologs, displayed by gene set. **(B)** A comparison between the mean and median orthologs of imprinted and sex differentiation gene sets.

Orthologs, evolutionarily related genes across different species that arose through speciation events, are generally considered to be one of the most competent measures of conservation from both functional and phylogenetic standpoints, over even paralogs 42. As shown in Figure 1, the SDGS maintained higher number of orthologs per gene by approximately 11%, by both mean and median measures, than the IGS. It also contained proportionately more individual instances of high ortholog number per gene compared to the IGS, with roughly 10% more genes in the 233-349/gene ortholog range and a corresponding 10% less genes in the 117-233/gene ortholog range. This infers that the IGS, despite its significant involvement in integral pathways like embryonic development, is less conserved than sex differentiation pathways like genitalia development.

### Synteny Conservation

**Figure 2:**
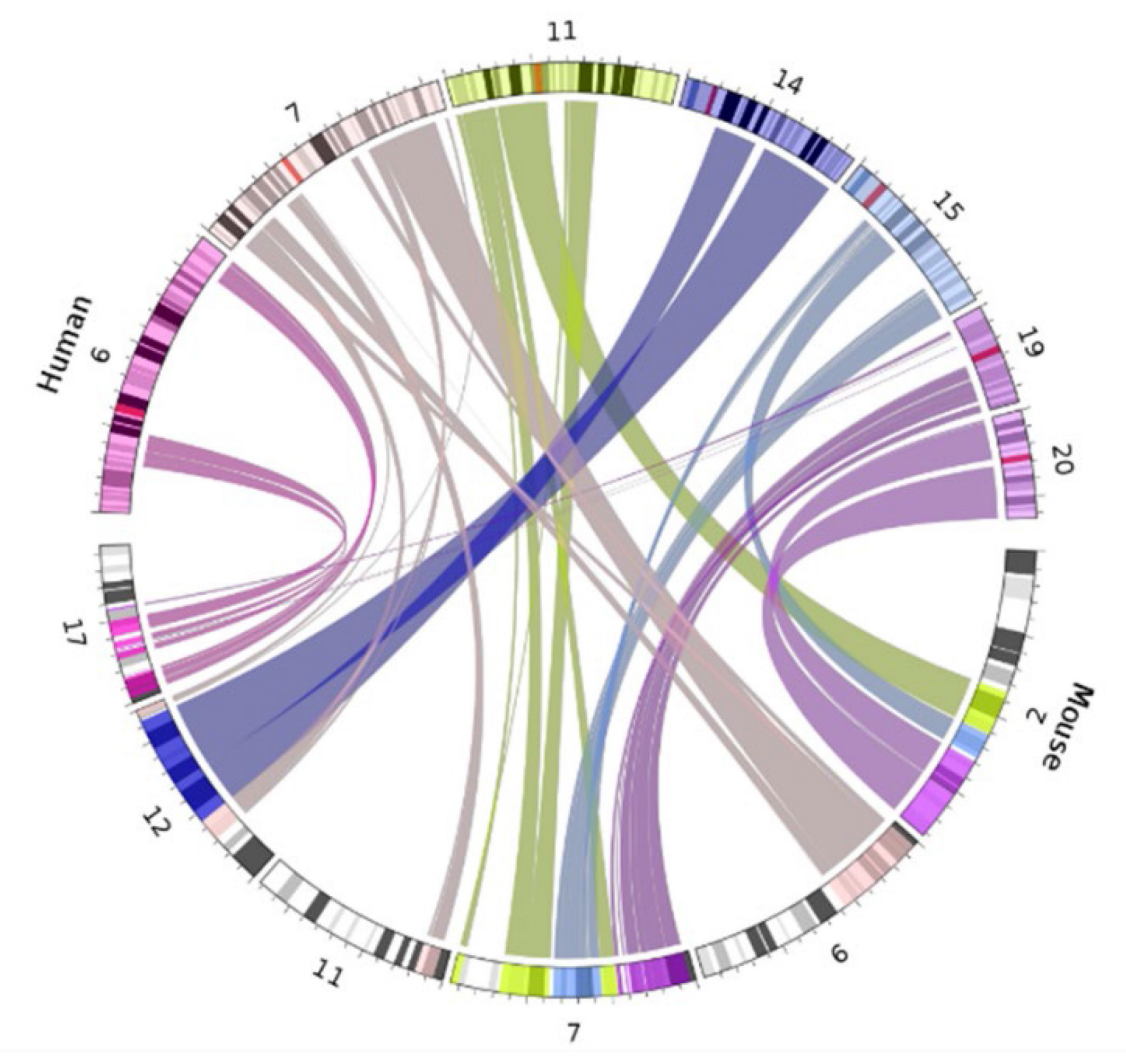
** Human and murine synteny.** Displays chromosomal synteny of imprinted genes between human and murine chromosomes.

Next we reviewed whether synteny played a role in imprinting status; if a significant amount of linkage disequilibrium was found between imprinted genes in closely related species that disruption could provide insight into why and how genes become imprinted. We looked at overlapping imprinted genes in human and murine genomes. Synteny looks for syntenic blocks, portions of the chromosomes with high linkage (gene clustering), between species, denoting a common ancestral region. Though traditional synteny does not measure gene order, more modern methods, like syntenic fragments, do measure it and this is referred to as collinearity 43,44. After analyzing the 75 genes overlapping between the human and murine imprinted gene sets (Figure 1) we determined that the syntenic clusters of these imprinted genes were highly conserved across the board, with the lowest number of conserved genes per cluster at 27 and the highest at over 500 (Figure 2 and Table 1) 45. Nearly every imprinted gene maintained the same immediate neighboring genes. Even in the rare instances when adjacent genes weren't identical, they were closely positioned within the cluster. Very high levels of collinearity were also observed in most clusters with the most common modification being inversion. There were two instances of translocations being the exception. We were only able to perform these analyses with protein coding genes as ncRNA genes are too poorly annotated and species-specific to provide meaningful data at this stage.

### Biotype Composition

**Figure 3:**
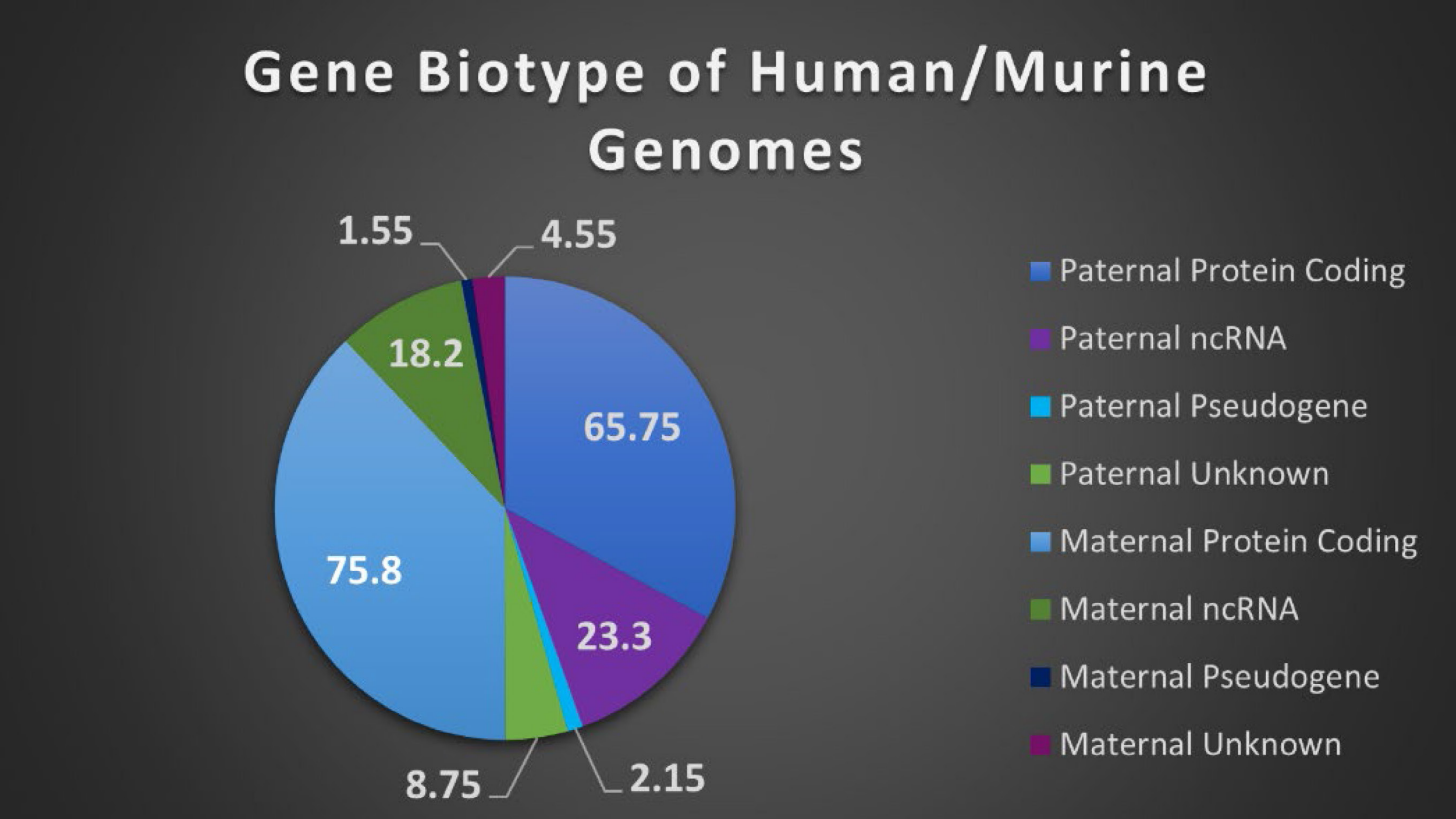
** Biotypes of human and murine imprinted genes.** A breakdown of biotypes (protein coding, pseudogene, noncoding RNA, or unknown) present in combined human/murine imprinted gene sets, separated by maternal or paternal expression.

The composition of biotypes in a gene set can be indicative of the role and pathways those genes comprise. Human and murine genome biotypes are predominately composed of protein coding genes, consistent with our current level of understanding and annotation of genes, and our IGS follows this convention with maternally expressed genes (MEGs) and paternally expressed genes (PEGs) comprised of approximately 76% and 66% protein coding biotypes (see Supplemental Table 1). Of note however, imprinted genes have a high level of ncRNAs, particularly long non-coding RNAs (lncRNAs), with MEGs at 18% and PEGs at 23% of total genes respectively. LncRNAs are categorized by a relative lack of protein homology, ORF size, and conservation with higher incidence of tissue specific expression and lower expression overall, when compared to protein coding genes 46. They are also highly correlated with expression of antisense genes, reinforcing the role of imprinted genes in the regulation (particularly in chromatin modification and transcriptional regulation) of tightly controlled biological pathways, with over 33% of the annotated ncRNAs comprising antisense transcripts (Figure 3 and Supplemental Table 1) 46,47.

### Imprinting Conservation

**Figure 4:**
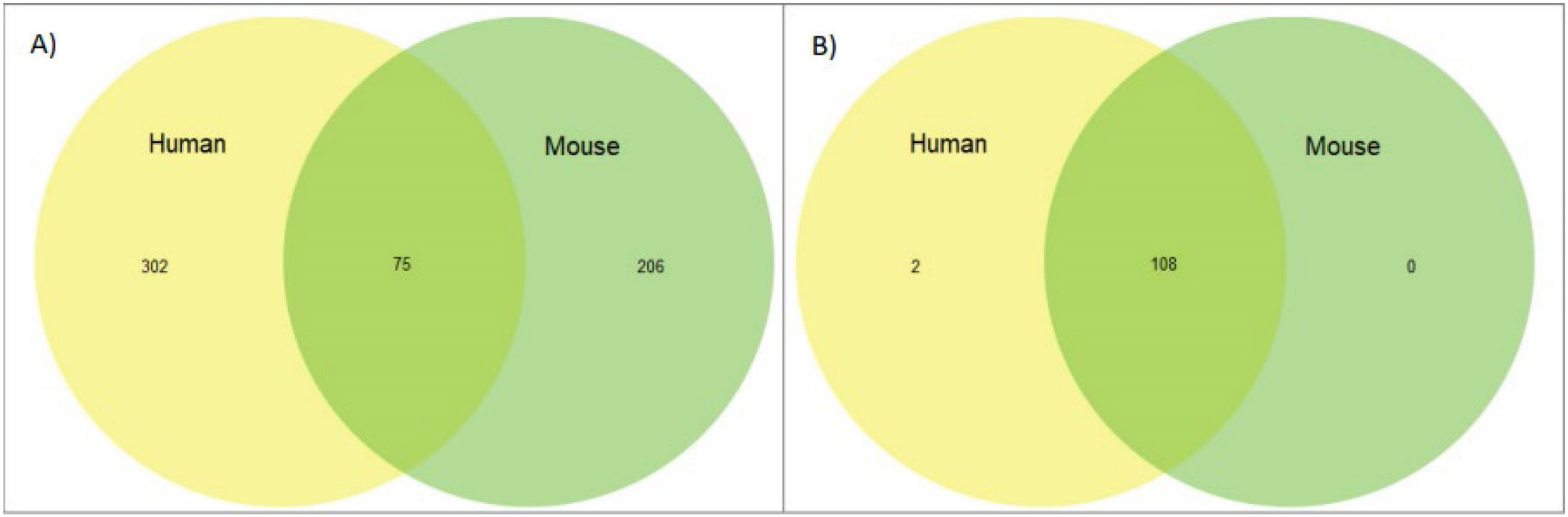
** Orthology of imprinted genes vs sex differentiation genes. (A)** Venn diagram of overlap between human and murine genes in the imprinted gene set.** (B)** Venn diagram of overlap between human and murine genes in the sex differentiation gene set.

Finally, we looked at the overlap of genes known to be imprinted between our two species versus whether those same genes are expressing imprinted status. We found that while genes that are known to be imprinted are highly conserved overall, the imprinting status of these genes is not, with the most significant overlap coming in the 75 genes shared between the human and murine genomes. This is significantly less overlap than demonstrated in the SDGS (Figure 4 and Supplemental Tables 1&2). Thirteen genes, *CDKN1C*, *DIO3*, *DLK1*, *GNAS*, *H19*, *IGF2*, *IGF2R*, *INPP5F*, *MEG3*, *MEST*, *PEG3*, *PHLDA2,* and *PLAGL1* are imprinted across multiple species and include the first discovered imprinted genes, often amongst the first genes explored when investigating imprinting status. These results can, in part, be attributed to the limited species and life stages of tissues that have been tested to this point and this overlap will most likely increase as the range of genes and species expands.

## Cross-Tissue Transcriptomics of Imprinted Genes

For the transcriptomics analysis we looked primarily at human RNA-seq data pulled from the NCBI database, due to both the scarcity of data available for non-human species sans mice and non-uniform expression categories between species, such as brain in humans and central nervous system (CNS) in mice. Some mouse expression data is also presented in growth stages, such as the CNS and limb tissues, which makes direct comparisons impossible. Here we compare expression data between IGS and SDGS, with values from 27 tissues normalized by reads per kilobase of transcript, per million or RPKM.

We established our own measure of expression, currently coined area, which serves as a highly accurate predictor (notably more precise than range) of both expression level across a range of tissue types and gene profiles (housekeeping versus tissue specific). This value is a percentile derived using the formula, where *n* is the number of tissues and *e* is the expression value of an individual tissue:



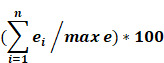



### Evolutionary Role

First, we looked at trends in imprinted gene expression on a tissue-by-tissue basis. In a broad sense, maternally expressed genes have been associated with the preservation of maternal resources and uniform allocation of available resources between offspring, in both nutrient allocation in the womb and maternal care postpartum. Paternally expressed genes are instead associated with securing more resources for their genetic offspring. The Sexual Antagonism (aka Intralocus Sexual Conflict), Maternal-Offspring Coadaptation, and Kinship theories have been proposed to explain why imprinting evolved 48-50. The Kinship theory is the most widely accepted and is supported by imprinted genes such as *IGF2*/*H19*. However, there are several genes that do not adhere tightly, or at all, to the Kinship theory which suggest multiple pressures led to the imprinting of sets or singletons of genes over time 51,52.

### Peak Tissue

**Figure 5:**
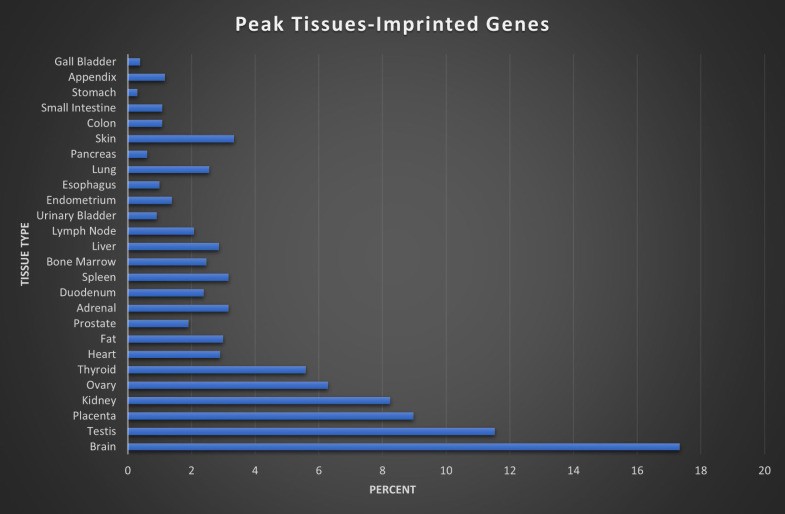
** Peak tissue types for imprinted genes.** Top tissue by gene abundance for the imprinted gene set (peak tissue is defined as the tissue in which the gene is most highly expressed). The highest peak tissue of the imprinted gene set by a significant margin is the brain. The imprinted gene set has high expression in the testis, with moderate expression in the placenta, kidney, and ovary tissues.

**Figure 6:**
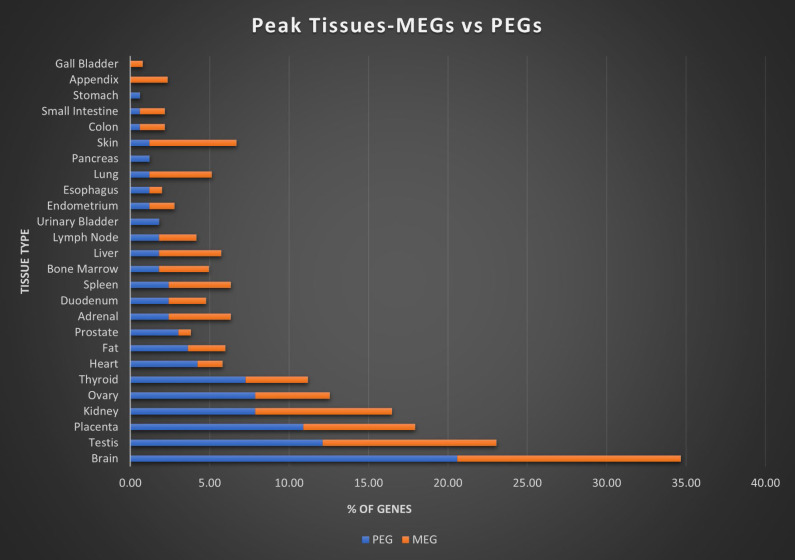
** Peak tissue types for MEGs vs PEGs.** Top tissue by gene abundance for the imprinted gene set (peak tissue is defined as the tissue in which the gene is most highly expressed). Both MEGs and PEGS have their highest expression in brain, testis, placenta, and kidney tissues. MEGs also show moderate expression in skin and ovary, with marginal to moderate expression in several others. PEGs show higher expression than MEGs in shared tissues and show expression in ovary and thyroid. MEGs have no peak tissues in stomach, pancreas, and urinary bladder tissues while PEGs have no peak tissues in the gall bladder or appendix.

Here we assigned a peak tissue to each gene according to expression abundance, with every tissue type represented except for the salivary gland, which didn't have the highest expressed gene of any gene in our IGS (Figure 5). The majority of MEGs and PEGs we investigated, had peak tissue levels in the brain, at approximately 18% of the total genes. Significant peak tissue levels also occurred in the testis and placenta (roughly 10% of total genes each). This supports the importance of imprinting in embryonic and brain development, as well as maternal behavior. Next, we explored the differences between expression patterns of both maternally and paternally IGSs (Figure 6). PEGs were not found in the gall bladder and appendix and were minimally expressed in the small intestine and colon (<1%). More than 5% of PEGs showed peak tissue levels in thyroid, ovary, kidney, placental, and brain tissues, at over 20% in the brain. Conversely, MEGs were not expressed in the stomach, pancreas, and urinary bladder, were marginally expressed in the prostate and gall bladder. Peak tissue levels of MEGs occurred in the skin, kidney, placenta, testis and especially the brain at nearly 15%. Overall, top tissue expression of MEGs was more evenly distributed across the various tissues examined when compared to PEGs. Combined these results seem to indicate that while imprinting effects are most closely linked to embryonic and neural development, they are active in nearly every tissue of the body.

### Inter-Set Differences

Several differences are evident between the imprinted set and the control set. Examination of the sex differentiation gene set showed a narrower range when displaying tissues of highest expression, with no peak tissues in the small intestine, colon, and heart in addition to salivary gland from the imprinted gene set (Figure S1). Sex differentiation genes have most peak tissue levels concentrated in the testis, with moderate presence in the skin, thyroid, ovary, kidney, endometrial, and brain tissues and only a minor placental role, indicating significant differences in the biological roles between these two gene sets despite the overlap in embryonic development and neural patterning. Next, we looked at the range of expression per tissue, here defined as the difference between the highest and lowest expression values of each respective gene, displayed between our imprinted and sex differentiation gene sets (Figure S2). In brief, the IGS showed overall higher ranges than sex differentiation genes, which could suggest that imprinted genes may be even more specialized. The range of imprinting genes was much higher in placental tissues (16% higher), and higher in liver and kidney (8 and 6% higher) tissues. Sex differentiation genes had much higher range in testis (10% higher), and higher in endometrium and adrenal (8 and 6% higher) tissues.

### Cross-Tissue Patterns

**Figure 7:**
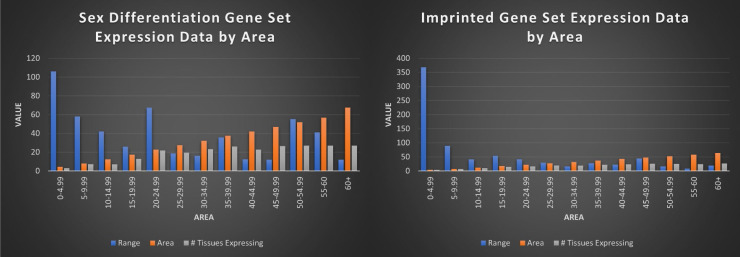
** Visualization of imprinted and sex differentiation gene sets by average range, area, and number of tissues expressed by area score.** This figure shows each area bracket (0-4.99, 5-9.99, etc) and the average range and number of tissues those genes are expressed in, for the genes in each bracket. For example, all imprinted genes with an area value of 0-4.99 have an average range of 368, average area of 4.25, and are expressed in an average of 3 tissues. Overall, the imprinted gene set is shown to be significantly more limited in tissues expressed, with higher range in low area genes, indicative of single or limited tissue genes.

Tested within our gene sets, our measure was able to consistently identify tissue specific genes (area values of < 3.8 at 100% single tissue expressing), genes expressed in limited tissues (area scores of 3.8-14.99 averaging <10 tissues expressing), and housekeeping genes profiles (area scores of 45+ averaging 27 tissues expressing). Combined with range values, we were able to quickly obtain reliable, high-level information about each gene in both gene sets regarding gene profile, with decreasing area scores indicating fewer tissues down to single tissue expression (the gene *PLG*) and increasing area scores leading to housekeeping (universal) expression, and the corresponding range score indicative of the strength of expression (the gene *RBL2*). Area score can even suggest relative levels of expression between tissues, where genes with low scores indicative of one highly (within the context of that gene's expression) expressed tissue and weak or very weak expression in other tissues expressing them. Conversely, genes with high area scores tend to have more even expression in many or all tissue types, with average values representative of a mix. When testing our gene sets, we considered minimum valid expression as 1 RPKM, with 5.1% of putatively imprinted genes and 1.8% of sex differentiation genes falling beneath this threshold 53. Both sets showed the highest representation in the 5-9.99 area bracket, with 60.7% of the imprinted gene set and 51.4% of the sex differentiation gene set in the 0-24.99 brackets. 9% and 6.4% of each set qualified as single tissue expressing genes, with 19% and 26% respectively following the housekeeping profile of ubiquitous expression (Figure 7).

### Pathway Enrichment

**Figure 8:**
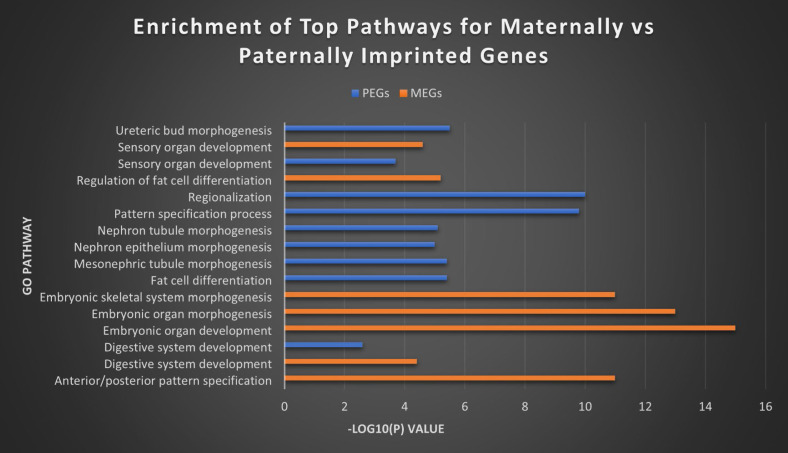
** Top MEG and PEG pathways.** The top separate and overlapping pathways for both maternally and paternally imprinted genes, by -Log10(P) value. MEGs were most strongly expressed in the morphogenesis and development of embryonic tissues, while PEGs showed their highest concentration in embryonic kidney morphogenesis and fat cell differentiation. They overlapped most significantly in digestive and sensory organ development.

In order to further investigate the relationship and roles of MEGs versus PEGs in biological pathways, we utilized the Metascape tool suite to perform gene ontology analysis 54. Although some overlap exists in the roles of both, substantial differences remain in the roles they fill in cell regulation and especially in development (Figure 8). MEGs displayed strong correlation with embryonic development/morphogenesis in organ and skeletal, and the creation of defined areas such as those in anterior/posterior specification and regionalization. PEGs meanwhile showed heavy involvement in kidney morphogenesis- renal tubule and metanephric nephron, development of the metanephric epithelium and regulation of the embryonic kidney, the mesonephros. They were also highly correlated with the regulation and differentiation of fat cells and the establishment of imprints during gametogenesis. The strongest overlap came in the development of Prader-Willi and Angelman syndromes, the development of sensory organs and digestive systems, developmental growth of a cell or organism, and cell regulation, including secretion and membrane potential.

### Inter-Set Pathway Enrichment

**Figure 9:**
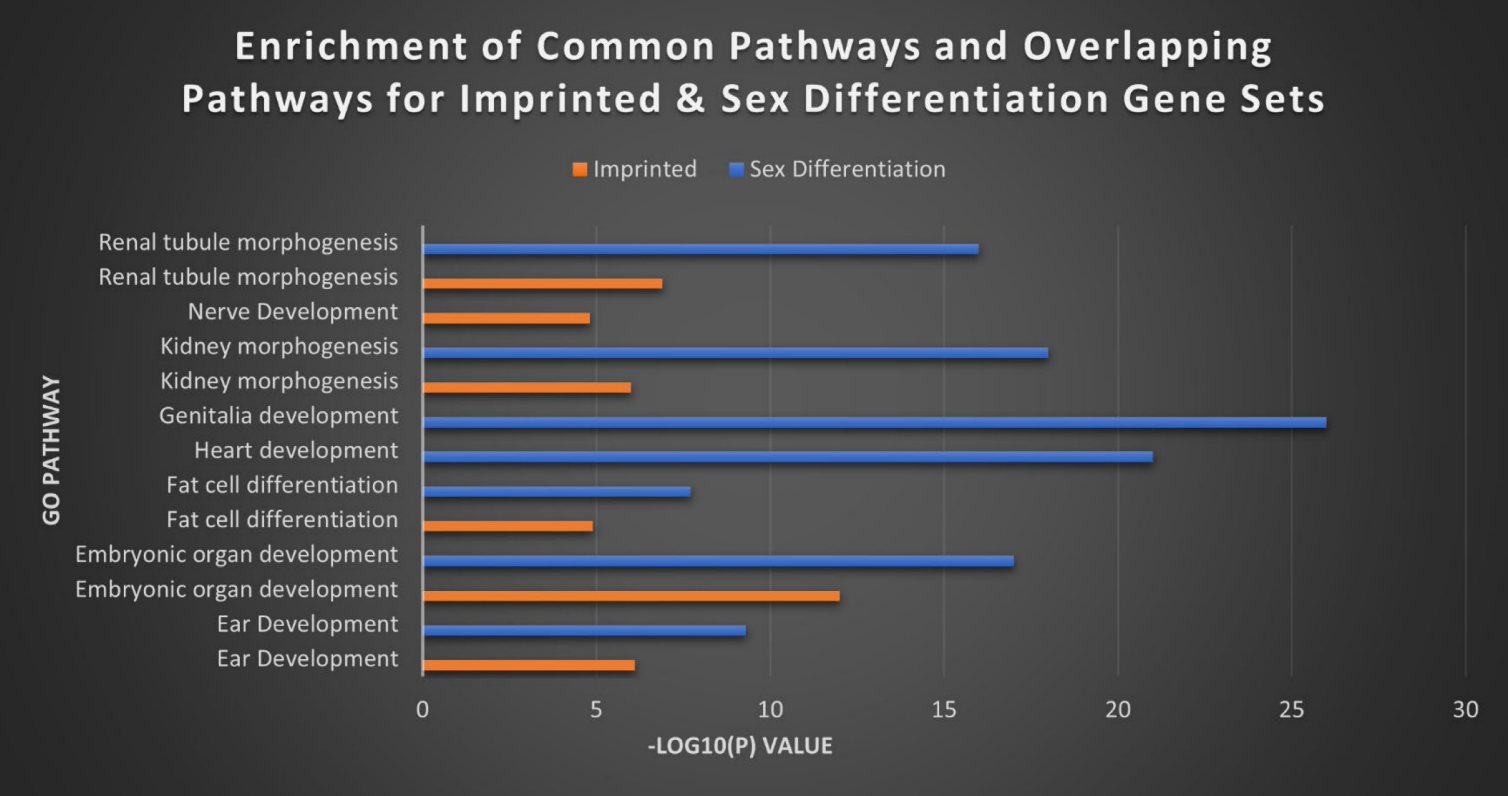
** Top imprinted and sex differentiation gene pathways.** The top separate and overlapping pathways of the imprinted gene and sex differentiation gene sets, by -Log10(P) value. The IGS were most significantly expressed in brain and nerve development, while the SDGS instead showed high expression in genitalia and heart development. The two sets overlapped in kidney morphogenesis, fat cell differentiation and ear development with the SDGS broadly exhibiting higher levels of expression overall.

Finally, we wanted to explore that same relationship between our imprinted gene set and what would appear to functionally be a very similar gene set in sex differentiation. GO analysis performed between the two sets revealed a high degree of overlap in areas like ear development, embryonic organ development, fat cell differentiation, kidney morphogenesis, renal tubule morphogenesis and urogenital system development. However, the SDGS displayed a broader range of biological processes than the seemingly more narrowly focused IGS (Figure 9). While broadly the IGS was more commonly associated with brain and nerve development, the SDGS were most strongly correlated with genitalia development, heart development, and general reproductive processes, among others. Heatmaps for the MEG versus PEG analysis and the imprinted versus sex differentiations sets can be found in Figures S3 and S4.

### Correlation

Studies of correlation within each gene set revealed overall higher levels of correlation within the SDGS, many moderately to strongly correlated, with notable absences in testis and placental tissues. The IGS showed overall slight to moderate levels of correlation inter-set with relative absence of correlation (none to slight) in placental, liver, duodenum, small intestine, stomach, and pancreas tissues with almost complete absence in salivary gland tissue. Both sets shared strong correlation in spleen, heart, fat, endometrial, and bladder tissues. Correlation figures can be found in Figures S5 and S6.

## Health Phenomics of Imprinted Genes

Even though imprinting only occurs in a very limited set of genes, it has been linked to a wide variety of diseases, including the category named after this phenomenon-imprinting disorders (ID). Some of these diseases are far ranging, as in the case of cancers which have been linked to ever widening networks of genes and causes, developmental disorders often caused by small aberrations in the precise timing and regulation of early morphogenesis, development, and differentiation, and mental and behavioral disorders that are broadly attributed to a complicated mix of personal and family genetics, trauma events, brain biochemistry, and perhaps most challenging- subjectivity 55. Though mental and behavioral disorders have traditionally languished in regards to both research and funding, they are beginning to get more attention and funding but still fall short of more traditionally focused fields, such as cardiovascular disease and cancer 56,57. Though narrower in scope in the context of people affected, IDs combine elements of both developmental and mental/behavioral disorders, making identification of their pathways difficult, and complicating and often delaying accurate diagnosis when a diagnosis can even be made 20,58.

### Gene Clustering and Disease Classification

**Figure 10:**
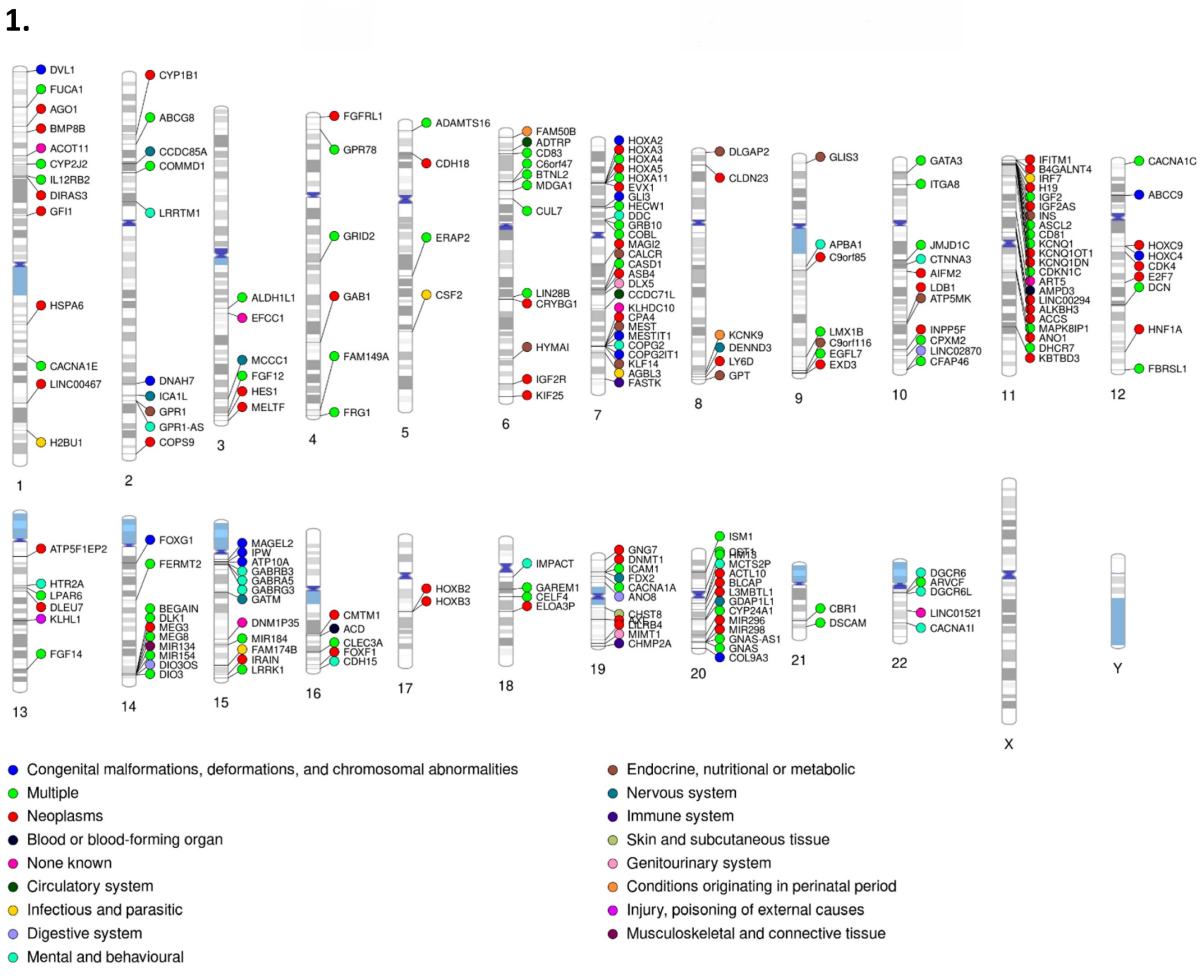

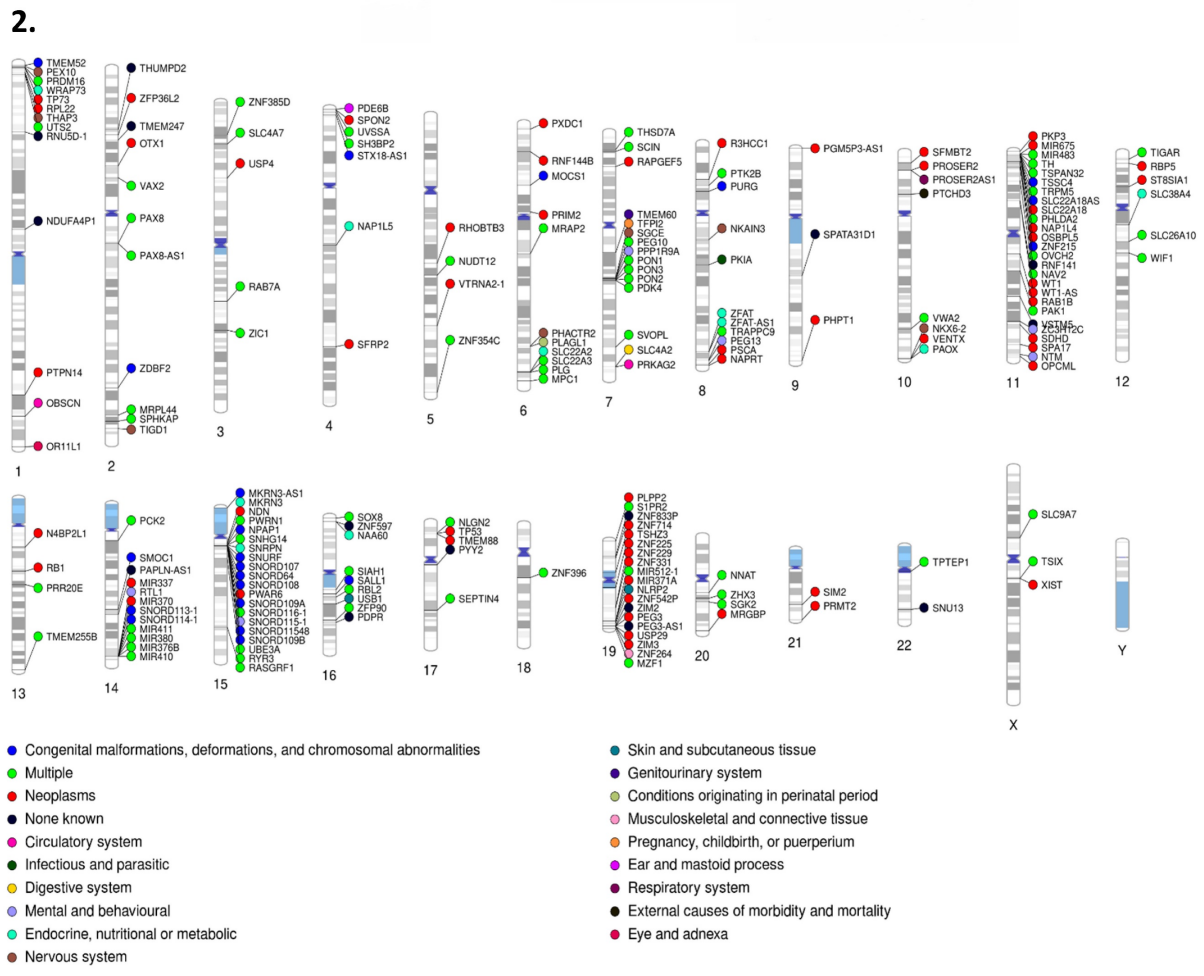
** 1. PhenoGram of human imprinted gene set (Part I). 2. PhenoGram of human imprinted gene set (Part 2).** Disease classification and chromosome location are displayed for each imprinted gene respectively. The dot color next to each gene indicates the category of disease that gene is associated with.

Our gene set had representation in every primary disease classification of the International Classification of Diseases, Tenth Revision (ICD-10) across all 22 autosomal chromosomes plus the X chromosome. Classification for each gene was determined by the disease related literature associated with that respective gene. For the disease classification figures (Figures 10-1 & 10-2), genes associated with multiple diseases were listed as multiple, outside of which each gene with the “multiple” disease classification counts as one instance of each associated disease. Disease percentages were determined by taking the total number of instances of each respective disease, divided by the total instances of disease, with the result multiplied by 100 as standard for percentiles. The most numerous disease categorization by far was neoplasms, masses formed by abnormal and uncontrolled cell growth, at 39.06% of total disease associations. The least numerous were external causes of morbidity and mortality, in this instance sensitivity to chemotherapy, and visual system at only 0.17%. Chromosomes 11 (13.5% of neoplasms and 10% of mental and behavioral disorders), 15 (almost 29% of congenital malformations and 16% of mental and behavioral disorders), and 7 (14% of congenital malformations), all well recognized sites of imprinting control regions and imprinting disorder risks, contained 9.93, 8.22, and 8.39% of genes. However, chromosome 1 (9% of endocrine disease), 6 (12.5% of endocrine diseases), and 14 (8.5% of congenital malformations), not typically recognized as centers of imprinting activity housed 7.02, 6.51, and 6.51% of genes respectively.

After we removed the nearly 40% of neoplasms to better judge the remaining disease phenotypes, we found that congenital malformations (17%), endocrine disease (~15%), mental and behavioral disorders (~14%), and nervous system disorders (~12%) account for nearly 59% of imprinting-associated disease classifications, with 5.8% of these genes currently lacking any known disease associations. After removing genes where the expressing allele was unknown or contested, MEGs comprised 194 individual instances compared to PEGs, which comprised 304. Of interest, PEGs had significantly higher presence in terms of number of associated genes in nearly every category, notably endocrine (~74%), mental and behavioral (~79%), and nervous system (~76%). The only category in which MEG genes were significantly more represented was in skin and subcutaneous tissues (~83%).

### Human-Murine Set Comparison

By comparison, the imprinted murine gene set also displayed its highest percentage as neoplasms (~43%) with the lowest, non-zero category being ear and mastoid process, injury-poisoning of external causes, and musculoskeletal and connective tissue at 0.72%. Removing neoplasms again, congenital malformations (~23%), nervous system disorders (~18%) endocrine diseases (~11%), and blood or blood forming organs/circulatory systems (~8% each) accounted for just over 67% of total disease classifications, similar to the human gene set though with a higher congenital and nervous system focus and subsequent reduction in mental and behavioral disorders and endocrine disorders. There were no known disease associations for 2.9% of this set (*Bbx, BC034090, Ifitm10, Pcdhb12*). PhenoGram visualizations reinforce the clustering nature of imprinted genes in both human and murine species, though the relatively remote location of some genes hints at the presence of more trans regulatory elements or as yet undiscovered imprinted genes or secondary DMRs 59. Both murine and sex differentiation PhenoGram images can be found in supplemental data (Figure S7 & S8). Disease classification across the IGS, SDGS, and murine sets can be found in Table 2.

### Inter-Set Disease Comparison

Finally, we compared our results to the SDGS. Unsurprisingly, neoplasms again had the highest percentage represented, with roughly 46% of the total, while the highest, non-zero category was tied between ear and mastoid process, infectious and parasitic, injury or poisoning of external causes, and respiratory system all at ~0.6%. This gene set was more evenly distributed, with chromosomes 2, 4, 5, and 10 containing 8.33, 7.14, 7.14, and 7.14% of the genes respectively with no significant concentrations of disease categories outside of neoplasms. This set had no representation in chromosome 15 but a much more notable presence on the X chromosome. While congenital malformations (~19%) and endocrine disease (~18%) remained high in this set, mental and behavioral disorders (~9%) and nervous system disorders (~4%) were associated at a significantly reduced rate. Instead, genitourinary system disease (~15%) and musculoskeletal and connective tissue disorders (~10%) were found at a much higher rate than either imprinted set (~5% or less for human/murine in genitourinary and musculoskeletal respectively) and rounded out the most highly represented categories in this set, with those four categories comprising 62% of disease phenotypes after neoplasms. Visualization of this set in PhenoGram shows more characteristic gene location, with some loose clustering indicative of traditional gene regulation rather than the highly clustered, localized control associated with imprinted genes. Both murine and sex differentiation PhenoGram images can be found in Figures S7 and S8.

## Limitations, Conclusion and Future Research

While assembling and analyzing this data a number of difficulties and limitations became apparent. First, there is wide disparity between sources and even between recently published papers in what genes are considered to be imprinted. We believe there are two primary reasons for these discrepancies. One is that imprinting in some genes can be tissue and even isoform dependent and has to be clearly delineated from uneven parental contributions, such as Y-linked genes, female genes that avoid X inactivation, and protein and mRNAs parental contributions to embryo cytoplasm 7. This has proven to be a difficult process. Second, many studies rely on tissue samples that are very limited, in terms of sample size, stage, and sex, even when considering non-human animal tissues. Their methods of detection are similarly varied and especially in non-human, non-murine animals there is a significant lack of overlap in gene verification, meaning that if a cattle gene was verified as imprinted 15 years ago it is likely that there has not been another attempt to confirm. Many genes have been identified for a high probability of imprinting but with no backing publications or directly contradictory results, likely stemming at least partially from what has been discussed here. All of this means the veracity of imprinting on some genes must be viewed as putative.

This lack of non-human, non-murine samples also made it impossible (at current time) to truly do an in-depth comparison or analysis of multiple species, outside of some basic gene and ortholog studies. Funding for imprinted gene research largely comes with a focus on human health, which is predominantly focused on human biological study through research models, such as mice and the rare human tissues. The existence of core imprinted genes, the 13 genes whose imprinted status has been confirmed in the most species, also limits in some ways the data available as the first imprinting studies in any animal invariably target these genes. As imprinting studies in these non-model animals are so rare, often being solitary or, at best, one of a handful of research papers, it limits the availability of data, even though it does contribute to overall knowledge concerning imprinting status. Our use of NCBI's gene database for expression data also limits the stage information available for our study.

Imprinted genes, while limited in abundance, are expressed in every tissue and across every chromosome with the exception of the Y chromosome. Though the genes themselves possess a significant number of orthologs, imprinting status does not appear to have a direct correlation, with at most 2-3% of orthologs maintaining the imprinted status, though they do show notably fewer orthologs than the comparable sex differentiation gene set, perhaps implying a lower rate of conservation consistent with the higher selection pressures and lack of redundancy in a system reliant on one allele. Synteny in imprinted gene sets between human and murine is strong, with very high collinearity and a relatively small number of significant chromosome events, such as translocations and relocations. While biotypes remain predominantly protein coding, a high percentage of ncRNAs, particularly lncRNAs, are present in both human and murine models which supports the mode of imprinted gene regulation and the regulatory role they are expected to play in the body.

Imprinted genes are typically found in thyroid, ovary, kidney, placenta, testes, and brain tissues, and are most abundantly expressed in placenta, kidney, liver, and brain tissues. Differences between MEGs and PEGs in these tissues were relatively negligible. Significant differences existed, however, in pathway activity. MEGs were strongly correlated to embryonic organ, cranial nerve, and digestive system development along with developmental growth, with their most significant pathways almost exclusively involved in embryonic morphogenesis, patterning, and development. PEGs were strongly correlated with fat cells, genomic imprinting, ureteric bud morphogenesis and sensory organ development, but their most significant pathways were focused almost entirely on morphogenesis and development of the embryonic and adult kidney, along with the establishment of imprinting marks. This is surprising in that, while imprinted genes have long been correlated with embryonic and neural development, there is a notable dearth of studies regarding imprinting activity or involvement in kidney tissues. Comparisons to the SDGS point to significant differences in distribution, range, and area, supporting the high degree of single or very limited tissue expression genes found in imprinting sets.

Limited correlation within the IGS against the SDGS supports this conclusion as does inspection of the most active pathways in each set, which showed the involvement of fewer genes at a higher incidence rate. The appearance of imprinted genes coincided with the evolution of placental birth in mammals, where the ability to fine-tune control of expression spatially and temporally beyond what could be provided in a system with two active alleles could provide significant evolutionary advantages, though that doesn't explain why it didn't evolve in reptiles who display the widest range of reproductive modes, including placentation.

In terms of disease phenotypes, both murine and human IGSs showed predictable focus in the categories of congenital malformations, endocrine disease, mental and behavioral disorders, and nervous system diseases which coincides with previous literature regarding imprinted gene function. Despite the prevalence of imprinted genes in the placenta however, only 1.93% (*FAM50B, GRB10, IGF2, KCNK9, MIR410, MRPL44, PLAGL*) and 3.31% (*DLK1, EGFL7, ERAP2, LIN28B, MIR184, MIR512-1, PEG10, PHLDA2, SIAH1, SNHG14, TFPI2, UTS2*), calculated after removal of neoplasms, of diseases were associated with the conditions originating in the perinatal period or pregnancy, childbirth, or puerperium categories. This may point to underrealized involvement of imprinted genes in pregnancy complications or to the long-term impacts on the fetus being recognized as congenital malformations, deformations, and chromosomal abnormalities instead. The skewed prevalence of PEGs and MEGs in disease categorizations may help future researchers narrow their focus when exploring imprinted gene involvement in disease mechanisms and preventative or ameliorative measures. The significant focus of PEG pathways on kidney morphogenesis and development, and lack of current literature, may also provide a promising direction for future research.

Our hope is that this collection and organization of genomic, expression, biological pathway, and disease phenotype data provides a valuable resource for the scientific community to explore and codify imprinted genes and their disease associations. Current understanding notes imprinting as the domain of placental mammals and endosperm bearing plants, but it's well documented association with behavior, including advanced maternal and nurturing behavior, leaves open the possibility for the discovery of imprinting in other species where this behavior occurs. For our part, we intend to explore the use and application of alternative transcripts and alternative polyadenylation sites in imprinted genes to determine extent of use and pattern recognition.

## Supplementary Material

Supplementary figures.

Supplementary table 1: Final Collection of Imprinted Genes.

Supplementary table 2: Final Sex Differentiation Genes.

## Figures and Tables

**Table 1:** ** Pairwise clusters and co-linearity.** Example of pairwise clustering in collinear imprinted genes, their species-specific chromosomes, and chromosomal alterations that occurred between species.

Genes	Human Chr	Mouse Chr	Pairwise genes/Cluster	Chromosomal Alteration
CALCR, DLX5, PEG10, PPP1R9A, SGCE, TFP12	7	6	27	None
COBL, DDC, GRB10	7	11	46	None
CDKN1C, IGF2, INS2, KCNQ1, PHLDA2	11	7	77	None
IGF2R, SLC22A2, SLC22A3	6	17	67	Inversion
PHACTR2, PLAGL1	6	10	228	Inversion
BLCAP, GNAS, NNAT	20	2	507	None

**Table 2:** ** Comparison of disease classifications by gene set.** Disease classifications by number of incidences, percentage of overall incidences that fall into this disease classification, and the percentage of overall incidences with neoplasms removed to allow for clearer demonstration of disease trends. All classifications were made per the International Classification of Disease, 10^th^ revision (ICD-10) by assigning categories per the disease literature available for each gene.

	Imprinted Genes- Human			Imprinted Genes-Murine			Sex Differentiation Genes-Human		
Disease classification	#	% of Overall	% No Cancer	#	% of Overall	% No Cancer	#	% of Overall	% No Cancer
Blood or blood-forming organ	5	0.84	1.38	6	4.35	7.59	0	0.00	0.00
Circulatory system	18	3.03	4.97	6	4.35	7.59	3	1.71	3.19
Conditions originating in perinatal period	7	1.18	1.93	0	0.00	0.00	0	0.00	0.00
Congenital malformations, deformations, and chromosomal abnormalities	61	10.27	16.85	18	13.04	22.78	18	10.29	19.15
Digestive system	12	2.02	3.31	2	1.45	2.53	3	1.71	3.19
Ear and mastoid process	4	0.67	1.10	1	0.72	1.27	1	0.57	1.06
Endocrine, nutritional or metabolic	56	9.43	15.47	9	6.52	11.39	17	9.71	18.09
External causes of morbidity and mortality	1	0.17	0.28	0	0.00	0.00	0	0.00	0.00
Eye and adnexa	6	1.01	1.66	2	1.45	2.53	0	0.00	0.00
Genitourinary system	13	2.19	3.59	4	2.90	5.06	14	8.00	14.89
Immune system	2	0.34	0.55	0	0.00	0.00	2	1.14	2.13
Infectious and parasitic	13	2.19	3.59	4	2.90	5.06	1	0.57	1.06
Injury, poisoning of external causes	4	0.67	1.10	1	0.72	1.27	1	0.57	1.06
Mental and behavioural	49	8.25	13.54	4	2.90	5.06	8	4.57	8.51
Musculoskeletal and connective tissue	19	3.20	5.25	1	0.72	1.27	9	5.14	9.57
Neoplasms	232	39.06		59	42.75		81	46.29	
Nervous system	44	7.41	12.15	14	10.14	17.72	4	2.29	4.26
None known	21	3.54	5.80	4	2.90	5.06	3	1.71	3.19
Pregnancy, childbirth, or puerperium	12	2.02	3.31	0	0.00	0.00	5	2.86	5.32
Respiratory system	4	0.67	1.10	3	2.17	3.80	1	0.57	1.06
Skin and subcutaneous tissue	7	1.18	1.93	0	0.00	0.00	2	1.14	2.13
Symptoms, signs, and abnormal findings not classified elsewhere	3	0.51	0.83	0	0.00	0.00	0	0.00	0.00
Visual system	1	0.17	0.28	0	0.00	0.00	2	1.14	2.13
