# Supplementary material for: Imprinted Genes: Genomic Conservation, Transcriptomic Dynamics and Phenomic Significance in Health and Diseases
Source: Int J Biol Sci. 2023 Jun 12;19(10):3128–42. doi: 10.7150/ijbs.83712 (PMC10321285; doi:10.7150/ijbs.83712)
Supplement: Supplementary file 1 — Supplementary figures. [file ijbsv19p3128s1.pdf]

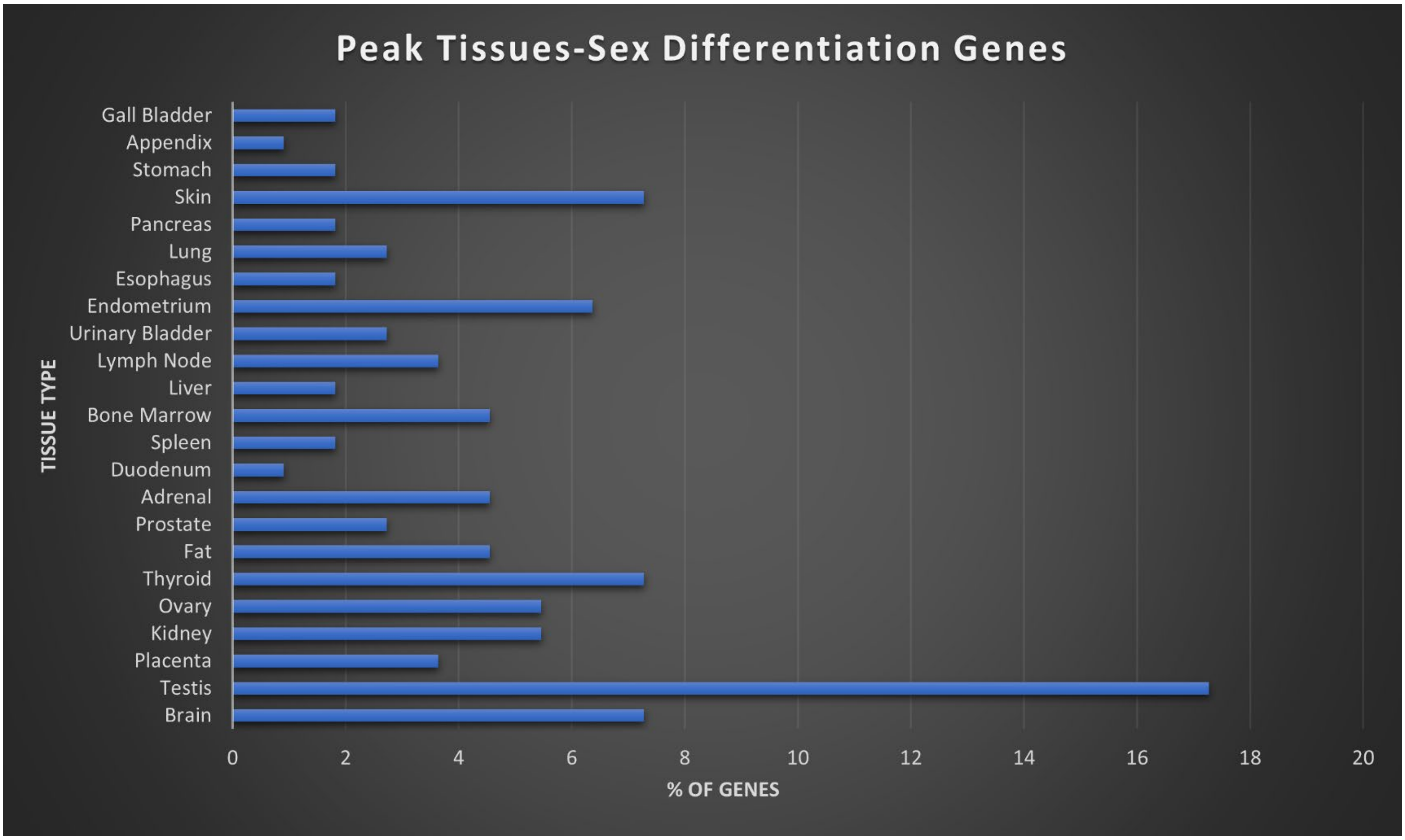

Figure S1. Peak Tissue Types for Sex Differentiation Genes
Top tissue by gene abundance for the sex differentiation gene set (peak tissue is defined as the tissue in which the gene is most highly expressed). The highest peak tissue by a significant margin is the testis. The sex differentiation gene set has moderate expression in skin, endometrium, thyroid, ovary, kidney and brain tissues.

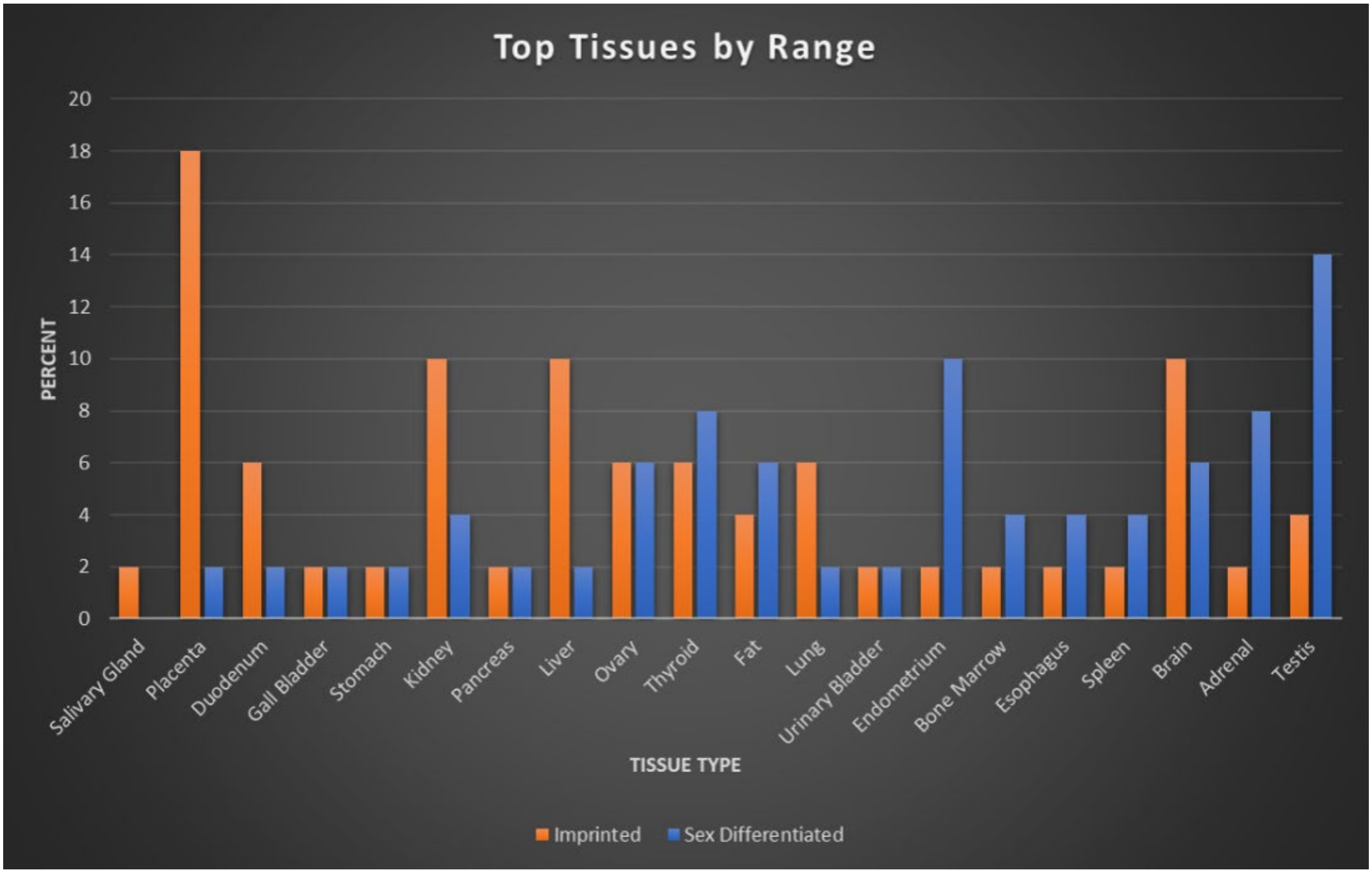

Figure S2. Comparison of Range By Tissue
Top tissues for each gene set by range. Range is determined by the difference between the minimum and maximum expression level across all tissues. High range scores are indicative of tissue-limited gene expression and can be indicative of expression levels. Overall imprinted genes demonstrate high range in placenta, kidney, liver, and brain, all of which have been associated with imprinting effects.

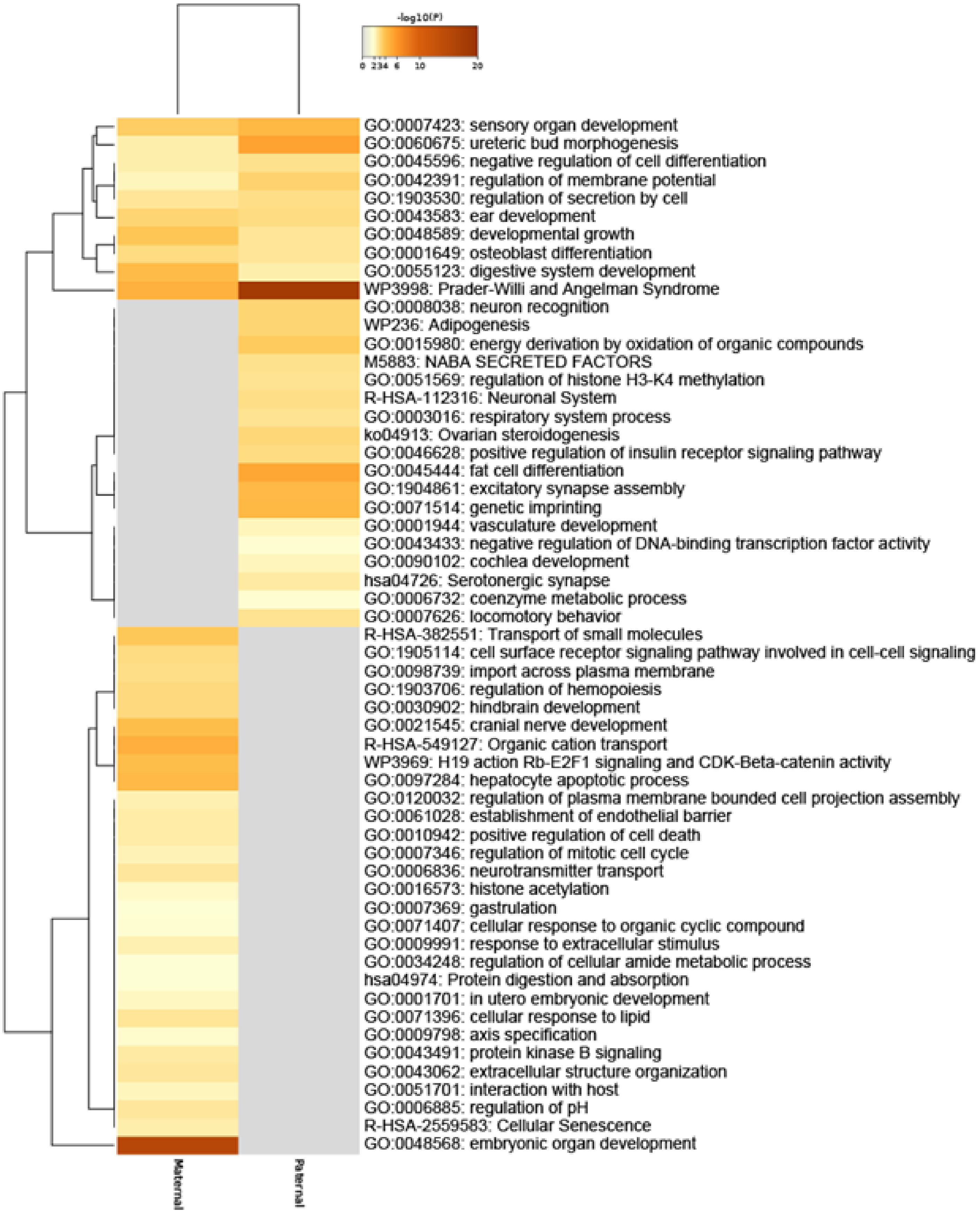

Figure S3. Top 100 MEG vs PEG Heatmap
Metascape heatmap of the MEG set vs the PEG set of imprinted genes.

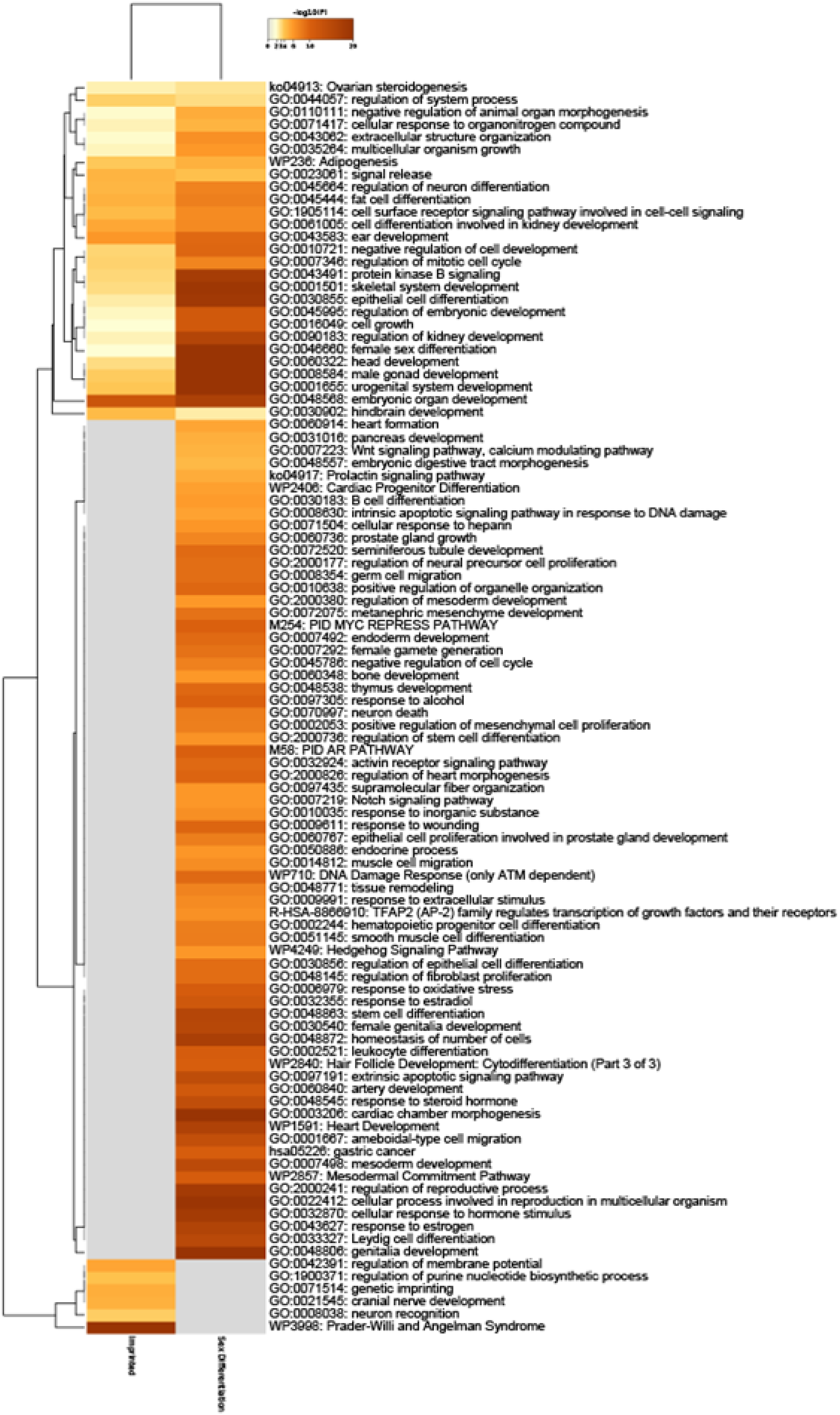

Figure S4. Top 100 Imprinted vs Sex Differentiation Heatmap
Metascape heatmap of the imprinted gene set vs the sex differentiation gene set.

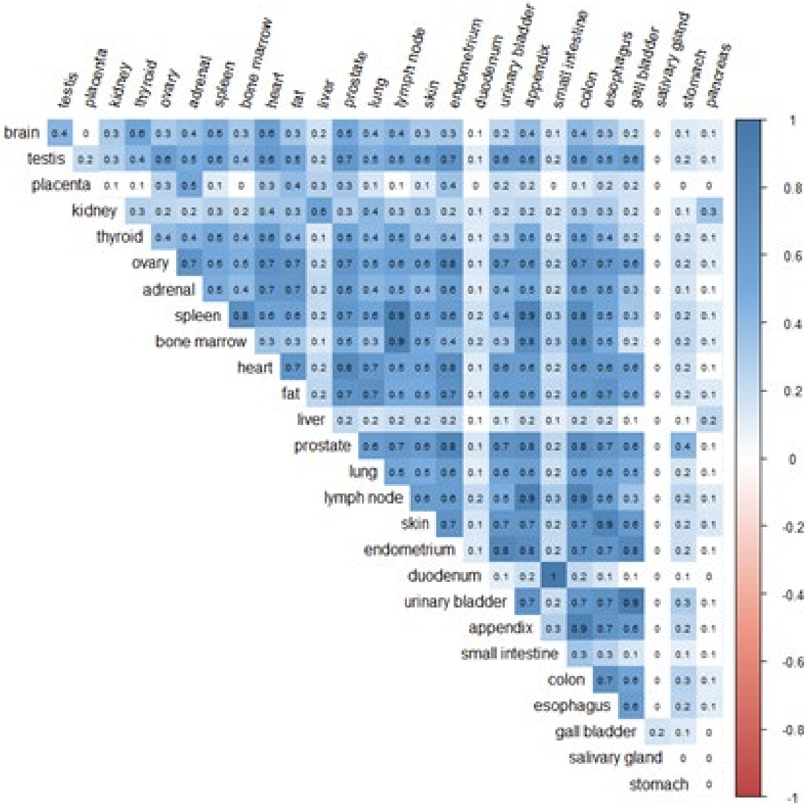

Figure S5. Gene Expression Correlation within the Imprinted Gene Set
A tissue correlation visualization of gene activity in the imprinted gene set.

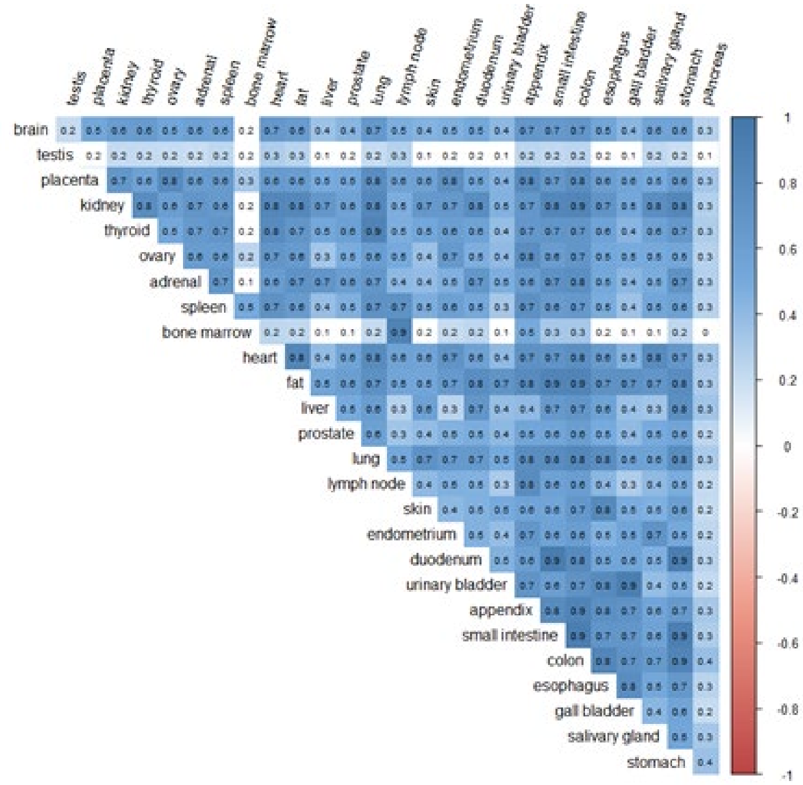

Figure S6. Gene Expression Correlation within the Sex Differentiation Gene Set
A tissue correlation visualization of gene activity in the Sex Differentiation gene set.

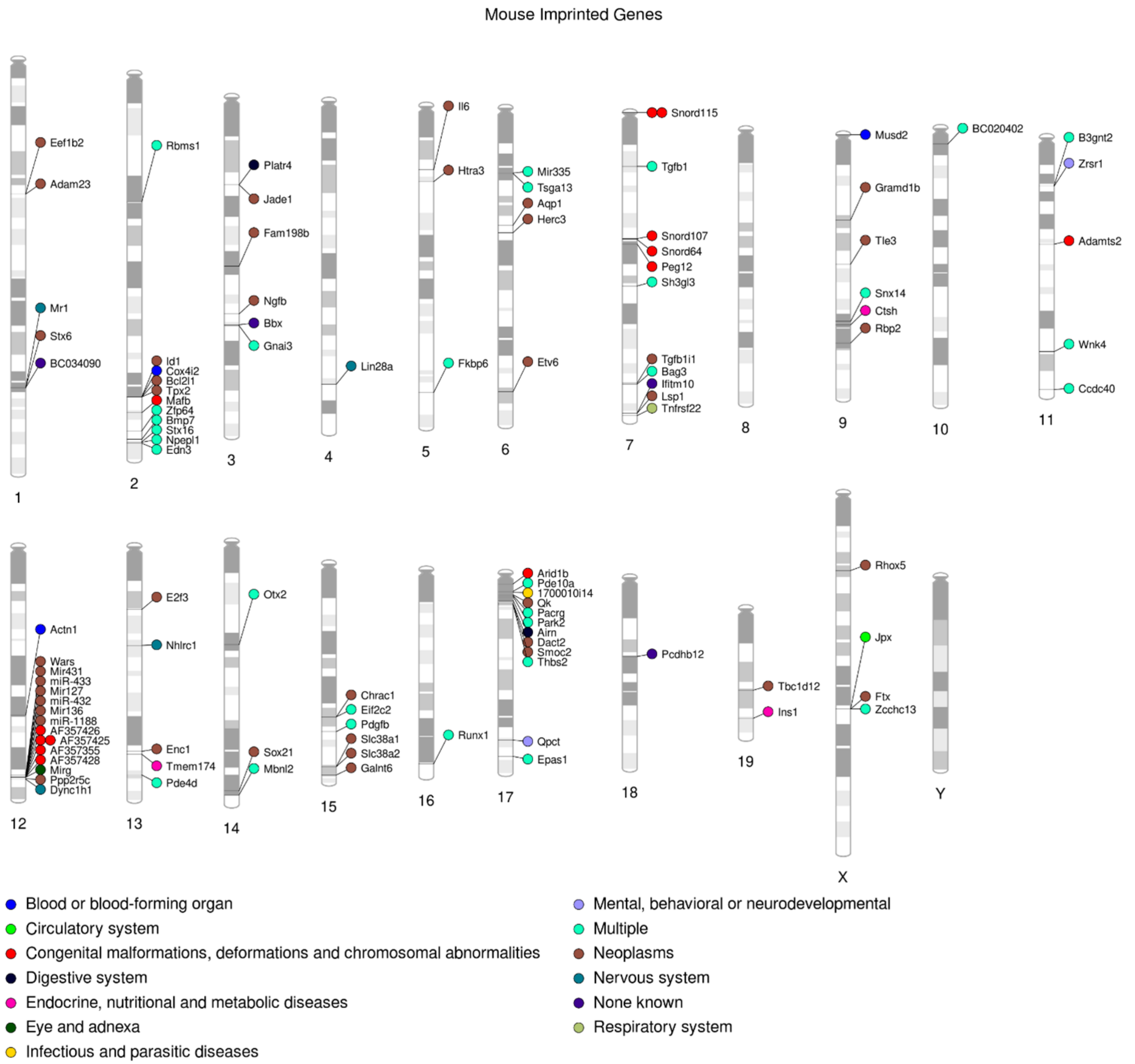

Figure S7. PhenoGram of Murine Imprinted Gene Set
Disease classification and chromosome location are displayed for each imprinted gene respectively. The dot color next to each gene indicates the category of disease that gene is associated with.

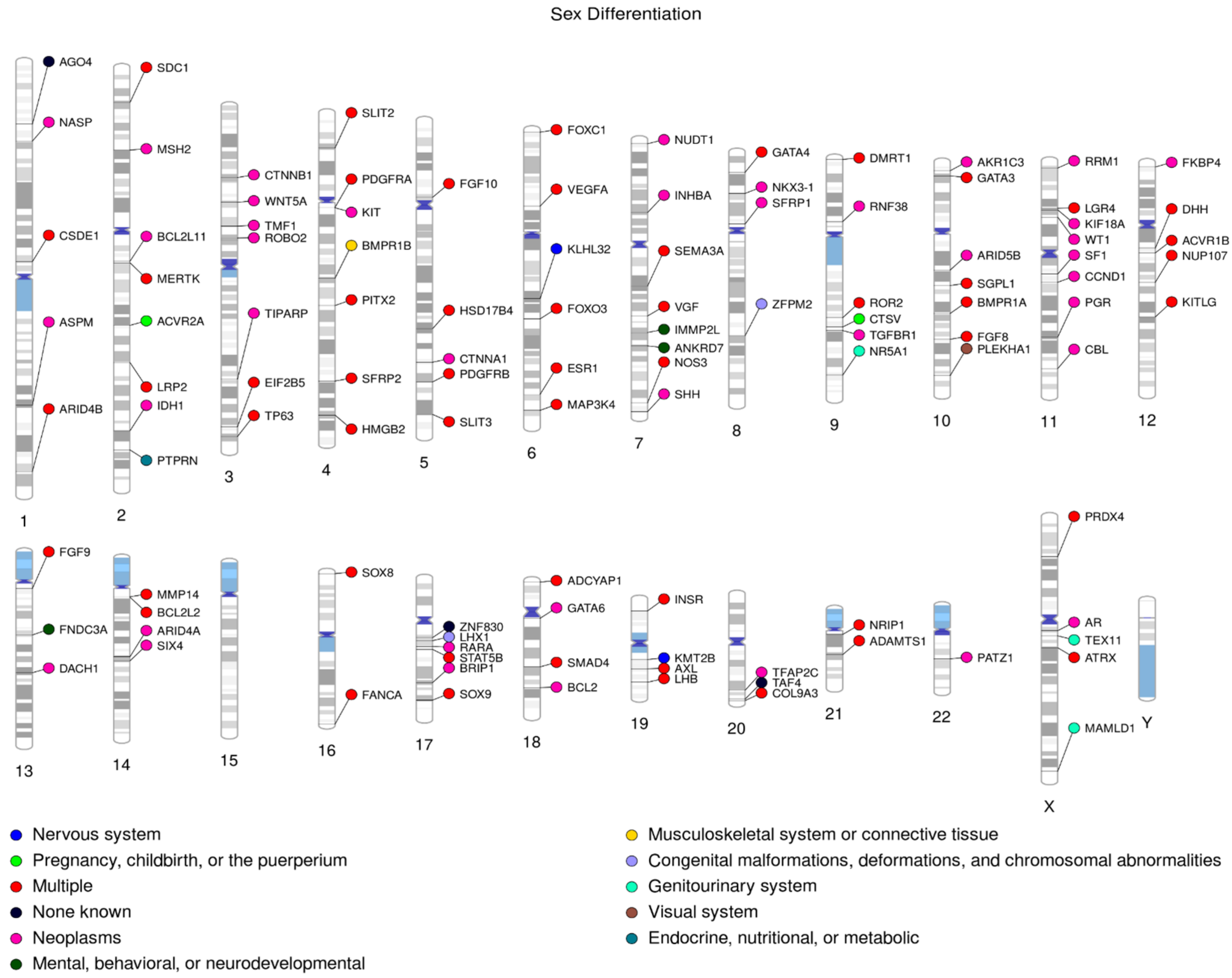

Figure S8. PhenoGram of Sex Differentiation Gene Set
Disease classification and chromosome location are displayed for each sex differentiation gene respectively. The dot color next to each gene indicates the category of disease that gene is associated with.
